# Pan-cancer analysis of non-oncogene addiction to DNA repair

**DOI:** 10.1038/s41598-021-02773-3

**Published:** 2021-12-01

**Authors:** Luis Bermúdez-Guzmán

**Affiliations:** 1 Robotic Radiosurgery Center, International Cancer Center, San José, Costa Rica; 2 Section of Genetics and Biotechnology, School of Biology, University of Costa Rica, San Pedro, San José, Costa Rica

**Keywords:** Cancer genetics, Tumour biomarkers, Cancer, Biomarkers, Oncology

## Abstract

Cancer cells usually depend on the aberrant function of one or few driver genes to initiate and promote their malignancy, an attribute known as oncogene addiction. However, cancer cells might become dependent on the normal cellular functions of certain genes that are not oncogenes but ensure cell survival (non-oncogene addiction). The downregulation or silencing of DNA repair genes and the consequent genetic and epigenetic instability is key to promote malignancy, but the activation of the DNA-damage response (DDR) has been shown to become a type of non-oncogene addiction that critically supports tumour survival. In the present study, a systematic evaluation of DNA repair addiction at the pan-cancer level was performed using data derived from The Cancer Dependency Map and The Cancer Genome Atlas (TCGA). From 241 DDR genes, 59 were identified as commonly essential in cancer cell lines. However, large differences were observed in terms of dependency scores in 423 cell lines and transcriptomic alterations across 18 cancer types. Among these 59 commonly essential genes, 14 genes were exclusively associated with better overall patient survival and 19 with worse overall survival. Notably, a specific molecular signature among the latter, characterized by DDR genes like UBE2T, RFC4, POLQ, BRIP1, and H2AFX showing the weakest dependency scores, but significant upregulation was strongly associated with worse survival. The present study supports the existence and importance of non-oncogenic addiction to DNA repair in cancer and may facilitate the identification of prognostic biomarkers and therapeutic opportunities.

Although tumours develop through a multistage process driven by the acquisition of genetic and epigenetic abnormalities, many of them become dependent on one or few genes to promote malignancy. As many of these genes were originally identified as oncogenes, this attribute was named oncogene addiction^1^. However, it is known that tumours can also become dependent on the normal cellular functions of certain genes, which themselves are not classical oncogenes, an attribute known as non-oncogene addiction (NOA)^2^.

In order to achieve uncontrolled proliferation, tumour cells rely upon the downregulation or epigenetic silencing of DNA Damage Repair (DDR) genes and the consequent increase in genetic and epigenetic instability^3^. Genome instability is indeed a fundamental hallmark of cancer^4^, possibly linked to oncogene-induced DNA damage^5^. Accordingly, it has been demonstrated that DDR genes alterations are prevalent in multiple human cancer types^6,7^. Despite the need for this genomic instability, tumours require some degree of DNA repair proficiency to survive the damage induced by genotoxic stress, uncontrolled proliferation, and treatments. Thus, the activation of the DNA-damage response in cancer can be considered as a type of NOA that critically supports tumour survival^8^. NOA genes are especially associated with stress maintenance functions such as DNA double-stranded break repair, chromatid segregation, and DNA replication regulation^9^. Accordingly, several DNA repair genes have been shown to exhibit Copy Number Variation (CNV) gain in cancer, which is positively correlated with upregulation of DDR genes^10^.

Given the importance of both oncogene and non-oncogene addiction, different groups have developed a variety of approaches to identify and prioritize cancer dependencies and vulnerabilities to exploit them therapeutically. The Cancer Dependency Map was initially created by systematically identifying genetic dependencies using RNAi-based loss-of-function genetic screens in 501 cancer cell lines^11^. Additionally, two large pan-cancer CRISPR-Cas9 screens were also independently performed with the same goal, containing data from over 1000 screens of more than 900 cell lines^12,13^. Based on both approaches, a combined CRISPR-shRNA dependency score was later developed, providing a more sensitive measure to identify essential genes^14^. These loss-of-function genetic screens have allowed investigating cellular drug mechanism-of-action in cancer cell lines^15^ as well as the identification of potential therapeutic targets like the DNA repair helicase WRN, a synthetic lethal vulnerability and promising target in cancers with microsatellite instability^16^.

In this study, I integrated data derived from The Cancer Dependency Map and transcriptomic data derived from The Cancer Genome Atlas (TCGA) to characterize the non-oncogene addiction to DNA repair across the pan-cancer scale. Next, I explored the relevance of DDR genes in terms of patient survival and potential therapeutic alternatives. Following this approach, a molecular signature of overexpressed DDR genes showing the weakest dependency scores was strongly associated with worse survival. The fact that cancer cells overexpress these genes despite the weak dependence on them (as growth promoters), suggests that they may act as late promoters of cell survival. Thus, as opposed to traditional cancer drivers, this supports the existence and importance of non-oncogenic addiction to DNA repair in cancer. This approach may facilitate the identification of prognostic biomarkers and therapeutic opportunities by targeting this non-oncogene addiction.

## Results

### Efficacy and selectivity of DNA repair genes

**Figure 1:**
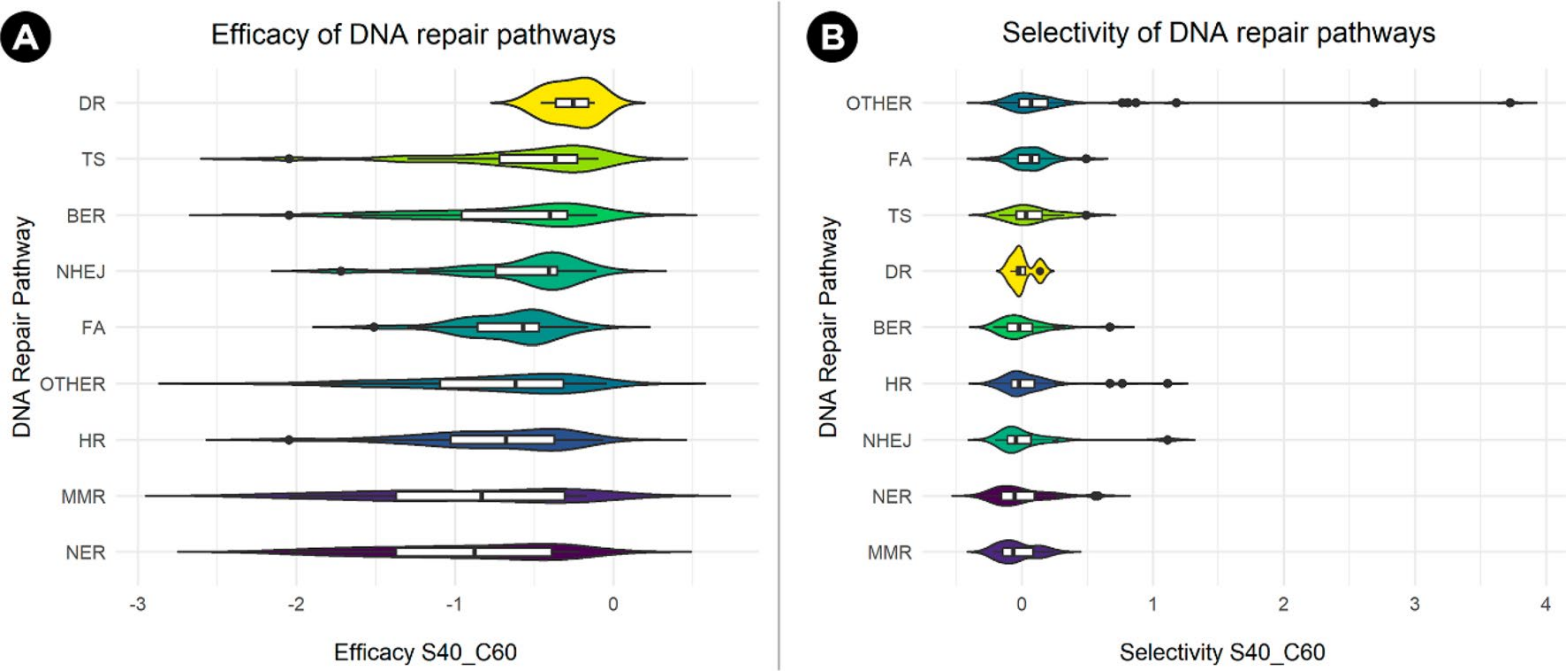
Efficacy and selectivity of DNA repair pathways. (**A**) Comparison of the efficacy scores (the more negative, the more essential) of the 241 DNA repair genes according to their pathway annotation (some genes are included in more than one pathway). (**B**) Comparison of the selectivity scores of the 241 DNA repair genes according to their pathway annotation (the more positive, the more selective). Notice that some pathways behave differently in terms of efficacy and selectivity. *DR* Direct repair, *TS* translesion synthesis, *BER* Base Excision Repair, *NHEJ* Non-Homologous End Joining, *FA* Fanconi Anemia, *OTHER* DNA repair-associated genes, *HR* Homologous Recombination, *MMR* Mismatch Repair, *NER* Nucleotide Excision Repair.

One of the few comprehensive lists of human DDR genes was published in 2005^17^. In order to obtain an updated list, a literature search was done and the most comprehensive list found was used as a starting point^6^. This list comprised 276 DDR genes annotated in ten different DNA repair pathways, but genes involved in the nucleotide pool maintenance were excluded in this study as that is not truly a pathway. First, to identify the essentiality of these genes across different types of cancer cell lines, the efficacy and selectivity scores (θ = 0.6; CRISPR:shRNA = 60:40; see methods) were obtained from shinyDepMap^14^. These metrics are derived from a combined CRISPR-shRNA gene dependency score based on the Cancer Dependency Map dataset^11^. Efficacy scores represent the degree to which loss of a particular gene reduces cell growth in sensitive lines while selectivity represents the degree to which its essentiality varies across lines. After removing genes that were not found in the shinyDepMap dataset, 241 DDR genes (Supplementary Table 1) were grouped in nine different DNA repair pathways and the efficacy scores for each pathway were compared (p = 0.02, *Kruskal–Wallis rank-sum test*, Fig. 1a). Although it seems that some pathways contain more genes with strongly negative efficacy scores, no statistically significant differences were found after Dunn’s Kruskal–Wallis Multiple Comparisons test. The same trend was observed for selectivity (p = 0.0004, *Kruskal–Wallis rank-sum test*, Fig. 1b).

**Figure 2:**
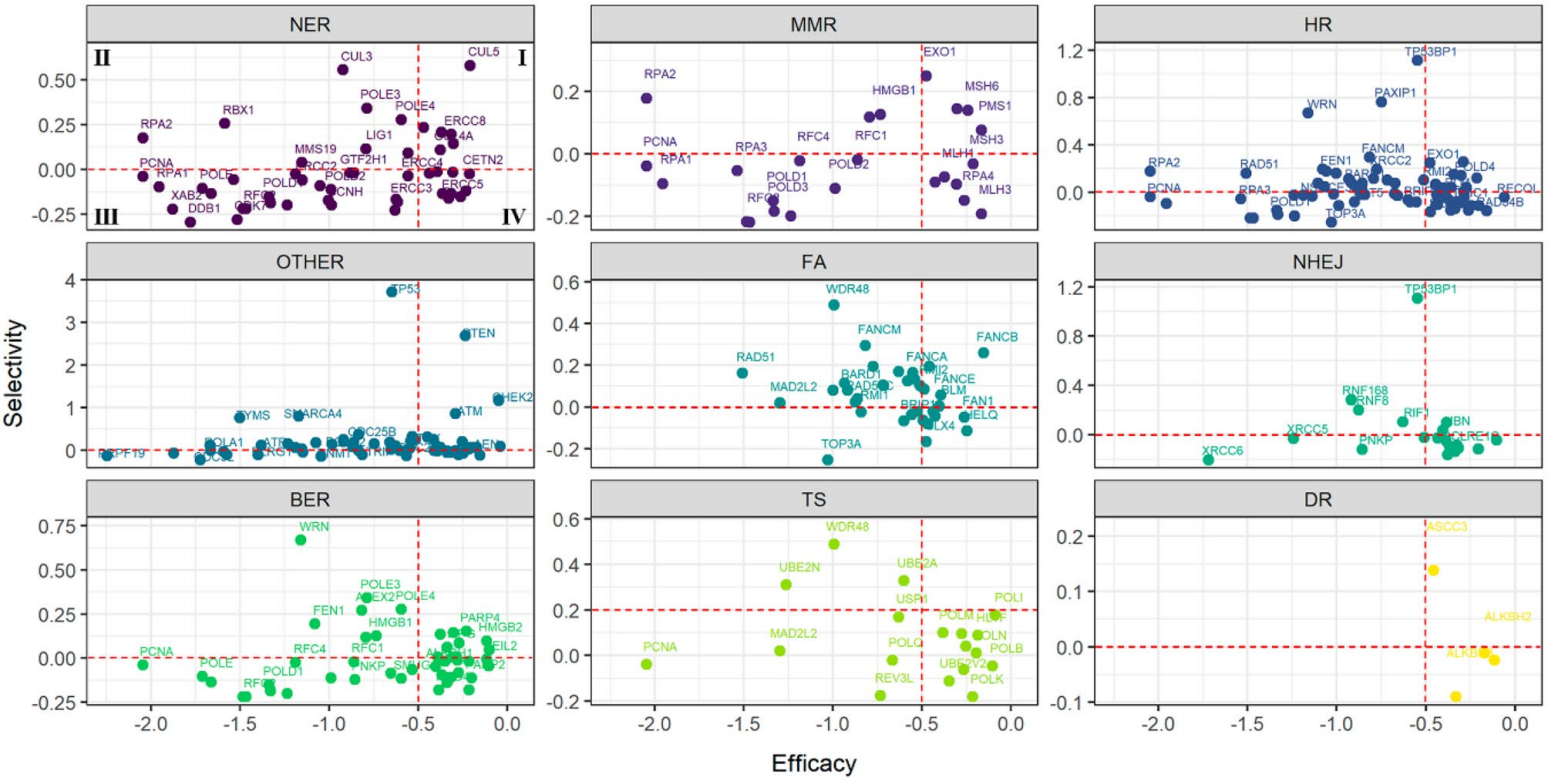
Correlation between selectivity and efficacy. The scatterplots show the correlation between efficacy and selectivity scores of the 241 genes grouped according to pathway annotation. Only NER showed a statistically significant positive correlation (*R* = 0.29, p = 0.045, *Spearman’s correlation test*). Genes with an efficacy score higher than − 0.5 and a selectivity score higher than 0.0 were discarded and only genes under the “essentiality threshold” (quadrant III) were selected and classified as “commonly essential genes” (n = 59). Notice that genes from quadrant II also have strongly negative efficacy scores but present high selectivity (e.g., HR/BER: *WRN*).

Next, as some pathways showed different behaviour in terms of efficacy and selectivity, the association between both metrics was determined. Genes were also grouped based on pathway annotation but only NER showed a statistically significant positive correlation (*R* = 0.29, p = 0.045, *Spearman’s correlation test*, Fig. 2). Additionally, to identify genes that are commonly essential in cancer cell lines (negative selectivity and negative efficacy scores), an “essentiality threshold” was set based on previous findings analyzing the whole set of human genes^14^. Following this approach, genes with efficacy scores higher than − 0.5 and selectivity scores higher than 0.0 were excluded. From the 241 genes initially included, only 59 were classified as commonly essential following these criteria (quadrant III, Fig. 2).

### Dependency scores of commonly essential DDR genes

**Figure 3:**
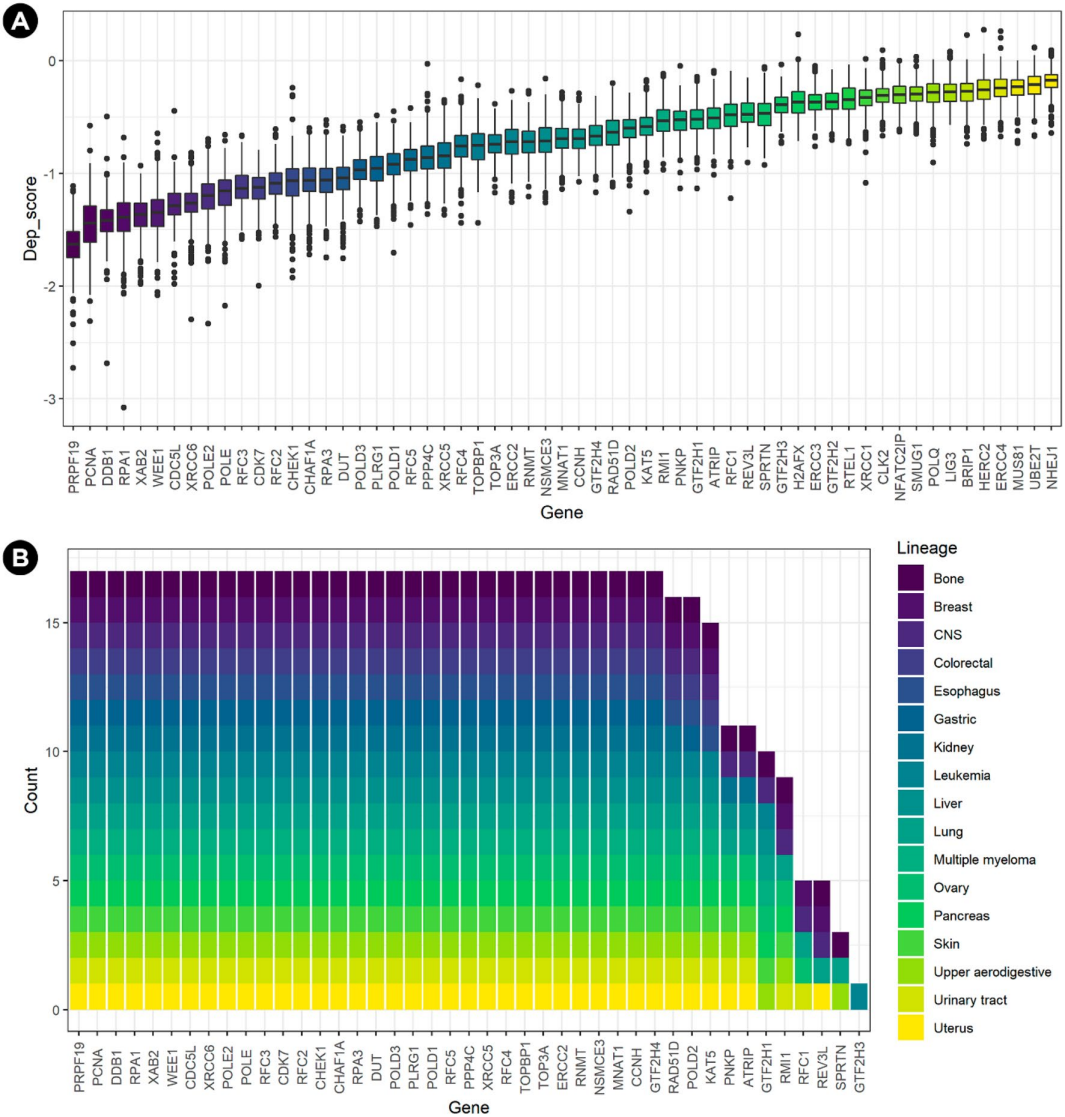
Combined (CRISPR:shRNA) dependency scores of 59 DDR commonly essential genes across 423 cancer cell lines. (**A**) The more negative dependency score, the more essential for a cell line. (**B**) When a gene has dependency scores beyond the threshold of essentiality in all the cell lines from a lineage, that lineage is dependent on that gene. All 17 lineages were dependent on 32 out of the 59 DDR genes.

To explore deeper the essentiality of these 59 DDR genes, the combined dependency scores (θ = 0.6; CRISPR:shRNA = 60:40) across 423 cancer cell lines were also obtained from the shinyDepMap dataset (Supplementary Table 2). Moreover, the lineage-dependent essentiality of 17 cell lineages was also analyzed. Only genes with at least one dependent lineage were included. Differences were found between these 59 genes in terms of dependency (p-value < 2.2e−16, *Kruskal–Wallis rank-sum test*, Fig. 3a). Only 43 of these genes had at least one dependent lineage (Fig. 3b), meaning that all cell lines for that lineage must have dependency scores lower than the essentiality threshold (near − 0.5) obtained by analyzing all human genes^14^. Notice that as expected, genes showing strongly negative dependency scores have more dependent lineages.

### Transcriptomic alterations of commonly essential DDR genes

**Figure 4:**
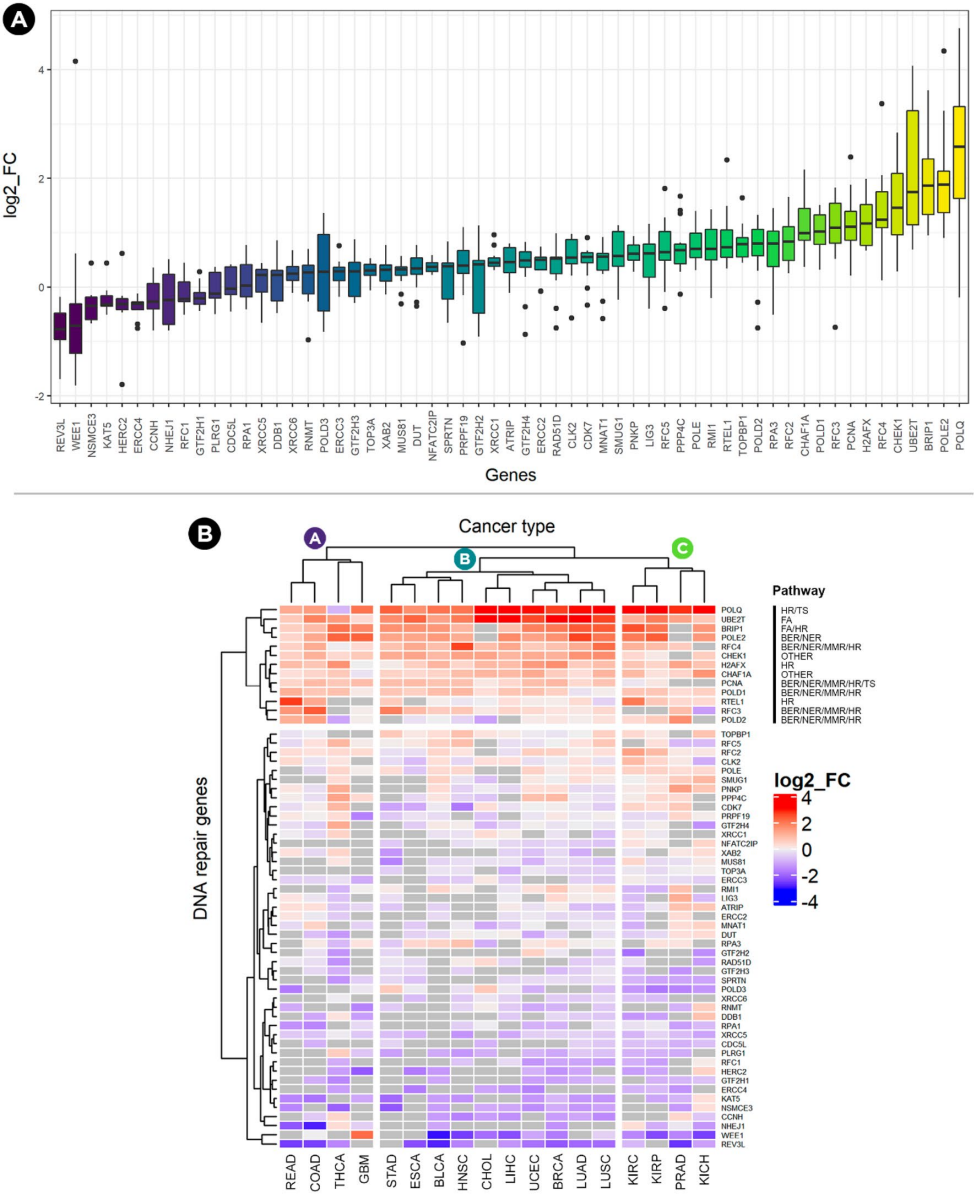
Log2 Fold Change of the 59 commonly essential DDR genes across 18 types of cancer. (**A**) Only genes with a log2 fold change with a statistically significant adjusted p-value were considered in each type of cancer. Notice that the more positive log2 FC, the greater the expression compared to normal tissue (**B**) Three clear groups are observed among all cancer types with at least 10 genes annotated in multiple pathways being highly upregulated (red colour) in most of them. No significant fold change is coloured in grey and downregulation in blue. Group A: READ: Rectum adenocarcinoma; COAD: Colon adenocarcinoma; THCA: Thyroid carcinoma; GBM: Glioblastoma multiforme. Group B: STAD: Stomach adenocarcinoma; ESCA: Esophageal carcinoma; BLCA: Bladder Urothelial Carcinoma; HNSC: Head and Neck squamous cell carcinoma; CHOL: Cholangiocarcinoma, LIHC: Liver hepatocellular carcinoma; UCEC: Uterine Corpus Endometrial Carcinoma; BRCA: Breast invasive carcinoma; LUAD: Lung adenocarcinoma; LUSC: Lung squamous cell carcinoma. Group C: KIRC: Kidney renal clear cell carcinoma; KIRP: Kidney renal papillary cell carcinoma; PRAD: Prostate adenocarcinoma; KICH: Kidney Chromophobe.

Since these 59 commonly essential DDR genes can also be essential for normal cells, the expression patterns of these genes were analyzed. For this purpose, the TACCO (Transcriptome Alterations in CanCer Omnibus) web server^18^ was used to obtain the log2 fold change (log2 FC) of these genes from paired normal-tumour samples. TACCO includes the mRNAs and miRNA expression levels (Transcripts Per Million) of 26 types of cancer from the Broad GDAC Firehose. Gene expression data were obtained for 18 types of cancer (Supplementary Table 3) after establishing two conditions: (1) only genes with a statistically significant log2 fold change (after p-value adjustment) were included and (2) only cancer types in which at least half of DDR genes meet condition 1 were included. Differences were also found between the 59 genes in terms of log2 FC, with some genes being downregulated and others highly upregulated (p-value < 2.2e−16, *Kruskal–Wallis rank-sum test*, Fig. 4a). Next, to have a better visualization of the expression levels of these 59 DDR genes across the 18 types of cancer, a heatmap was elaborated with the log2 FC of each gene (Fig. 4b). Three groups are observed among the cancer types with at least 10 genes being highly upregulated in most of them. The majority of these genes are part of several pathways or participate in Homologous Recombination. For many genes either there is no significant fold change (grey colour), or they are downregulated.

**Figure 5:**
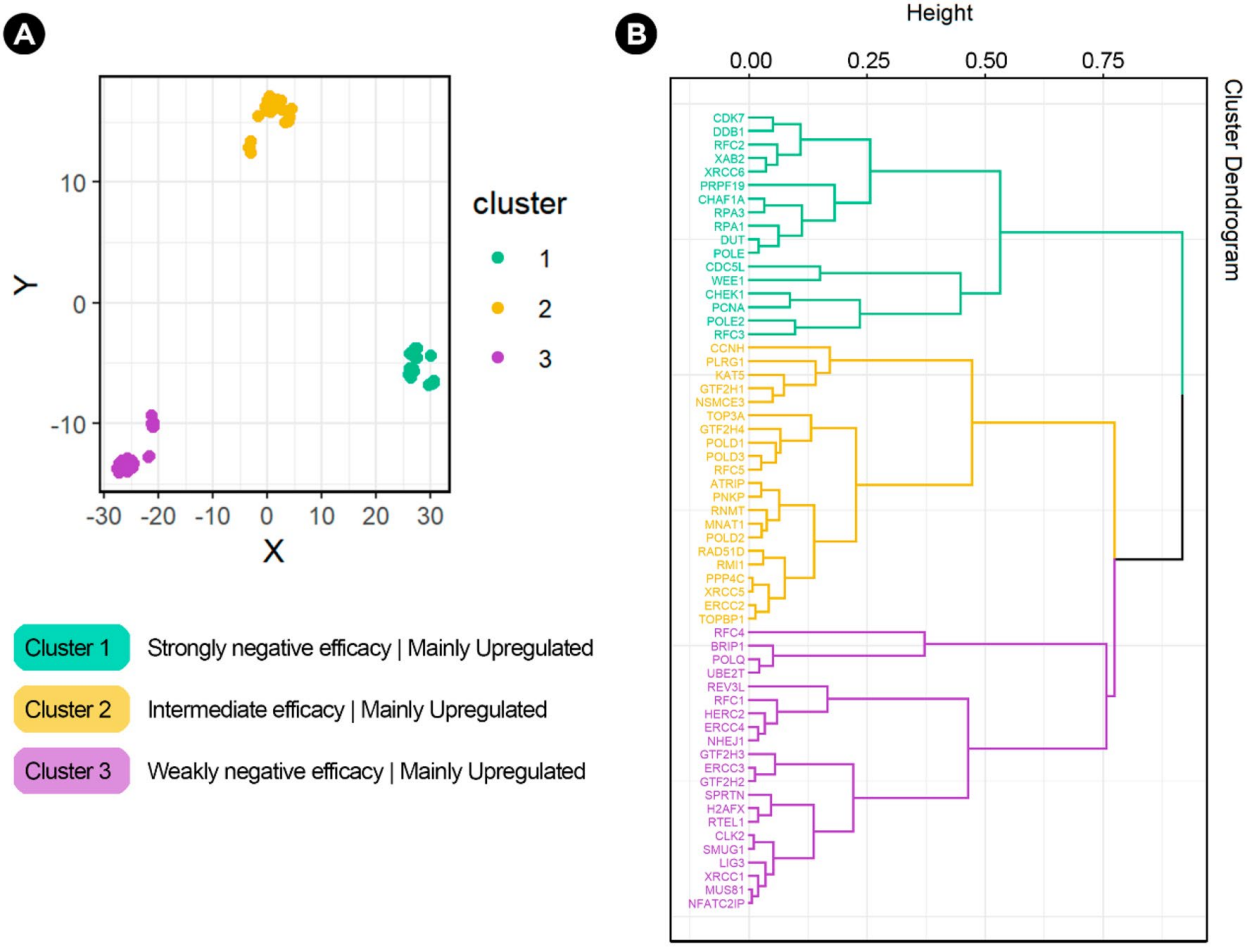
Clustering of the 59 DDR commonly essential genes. Gower’s similarity coefficient was used to calculate the distance matrix based on efficacy, selectivity, dependency scores, and log2 FC of the 59 commonly essential DDR genes. (**A**) Partitioning Around Medoids (PAM) was used as a clustering algorithm and the t-Distributed Stochastic Neighbor Embedding (tSNE) algorithm was used for dimensionality reduction. Note that the size, distance, and shape of clusters in the tSNE analysis do not always convey a meaning as clusters may vary upon initialization, perplexity values, and iteration. (**B**) Hierarchical clustering for dimensionality reduction based on the distance matrix calculated using Gower’s distance and the hclust algorithm.

Since it was noticed that genes with strongly negative dependency scores (more important for cancer cell growth) were not always highly upregulated, all variables were analyzed together to further explore clustering patterns. The efficacy and selectivity scores were included as continuous variables. The dependency scores of the 59 genes (for the whole set of 423 cancer cell lines) and the log2 FC (for the 18 types of cancer) were transformed from continuous to categorical (ordinal) variables (see methods) (Supplementary Table 4). A distance matrix was created based on Gower’s similarity coefficient. Three clusters were generated using the Partitioning Around Medoids (PAM) and hclust algorithms. The t-Distributed Stochastic Neighbor Embedding (tSNE) algorithm (perplexity = 10, max. iteration = 1000) and Hierarchical clustering were used for dimensionality reduction. Figure 5 shows the three clusters generated based on the two clustering algorithms. Interestingly, cluster 1 consists of upregulated genes with strongly negative dependency and efficacy scores. On the contrary, cluster 3 consists of upregulated genes but they all have the weakest negative dependency and efficacy scores. Cluster 2 consists of genes with intermediate scores.

### Commonly essential DDR genes survival analysis

**Figure 6:**
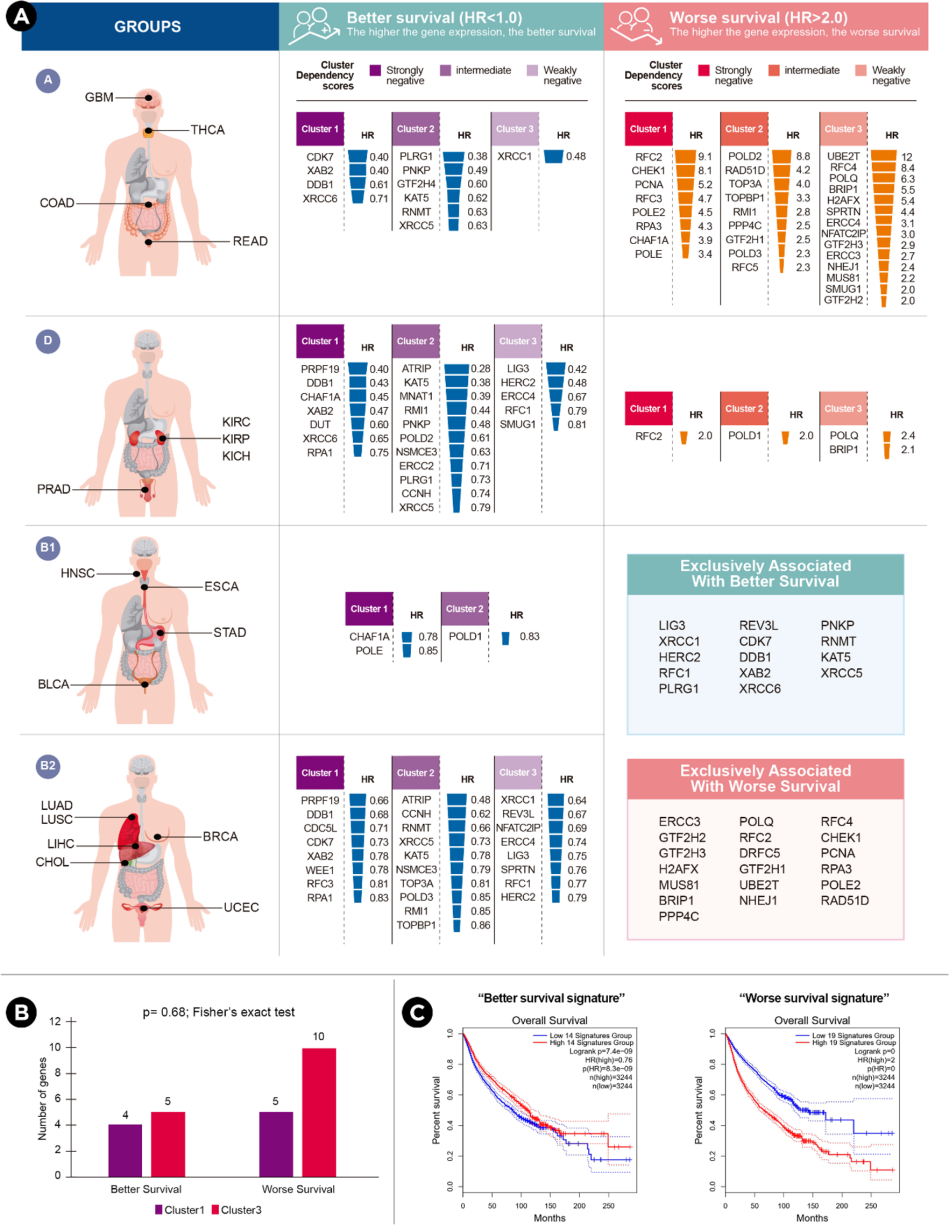
Survival analysis based on the expression levels of the 59 commonly essential DDR genes. (**A**) Genes whose higher expression was associated with better or worse overall survival (with a statistically significant log-rank P-value and statistically significant hazard ratio HR > 2/HR < 1) are indicated for each cancer group. Genes exclusively associated with the better/worse survival signature are indicated. (**B**) No statistically significant differences were found between the number of genes contributed by clusters 1 and 3 to each signature (p = 0.68, *Fisher's exact test*). (**C**) Prognostic value of the “better/worse” signatures in terms of overall survival (considering the 18 cancer types together).

To explore the clinical significance of the above findings, the GEPIA (Gene Expression Profiling Interactive Analysis) web server^19^ was used to perform survival analysis based on gene expression levels. GEPIA delivers fast and customizable functionalities using The Cancer Genome Atlas (TCGA) dataset^20^. The genes from each cluster were analyzed to determine if their expression was associated with worse or better overall survival in each of the three cancer groups (A, B, C) from the heatmap above (group B was subdivided into B1 and B2 as it was much larger and heterogeneous than A and C). Almost all 59 genes were statistically significant associated with worse or better survival at least in one cancer group (Supplementary Table 5). However, only genes whose higher expression was associated with a statistically significant log-rank P-value and statistically significant hazard ratio (HR > 2/HR < 1) were selected for further analysis (Fig. 6a).

As several genes have opposite effects for two or more cancer groups, genes that were exclusively associated with better survival and those exclusively associated with worse survival were analyzed as separate molecular signatures. Fourteen genes were classified as “better survival” (four from cluster 1 and five from cluster 3) whereas nineteen genes were classified as “worse survival” (five from cluster 1 and ten from cluster 3). Despite this trend, no statistically significant differences were found between the number of genes contributed by clusters 1 and 3 to each signature (p = 0.68, *Fisher's exact test*; Fig. 6b). The “better survival” gene signature was analyzed in terms of overall survival and as expected, it was associated with better survival across the 18 cancer types included in this study (p = 7.4e−09; HR 0.76; pHR = 8.3e−09, n = 3244; Fig. 6c). On the contrary, the “worse survival” gene signature was associated with worse overall survival (p = 0; HR 2.0; pHR = 0, n = 3244; Fig. 6c). Notice that p values of cero must be understood as “below machine precision” (see “Methods”).

**Figure 7:**
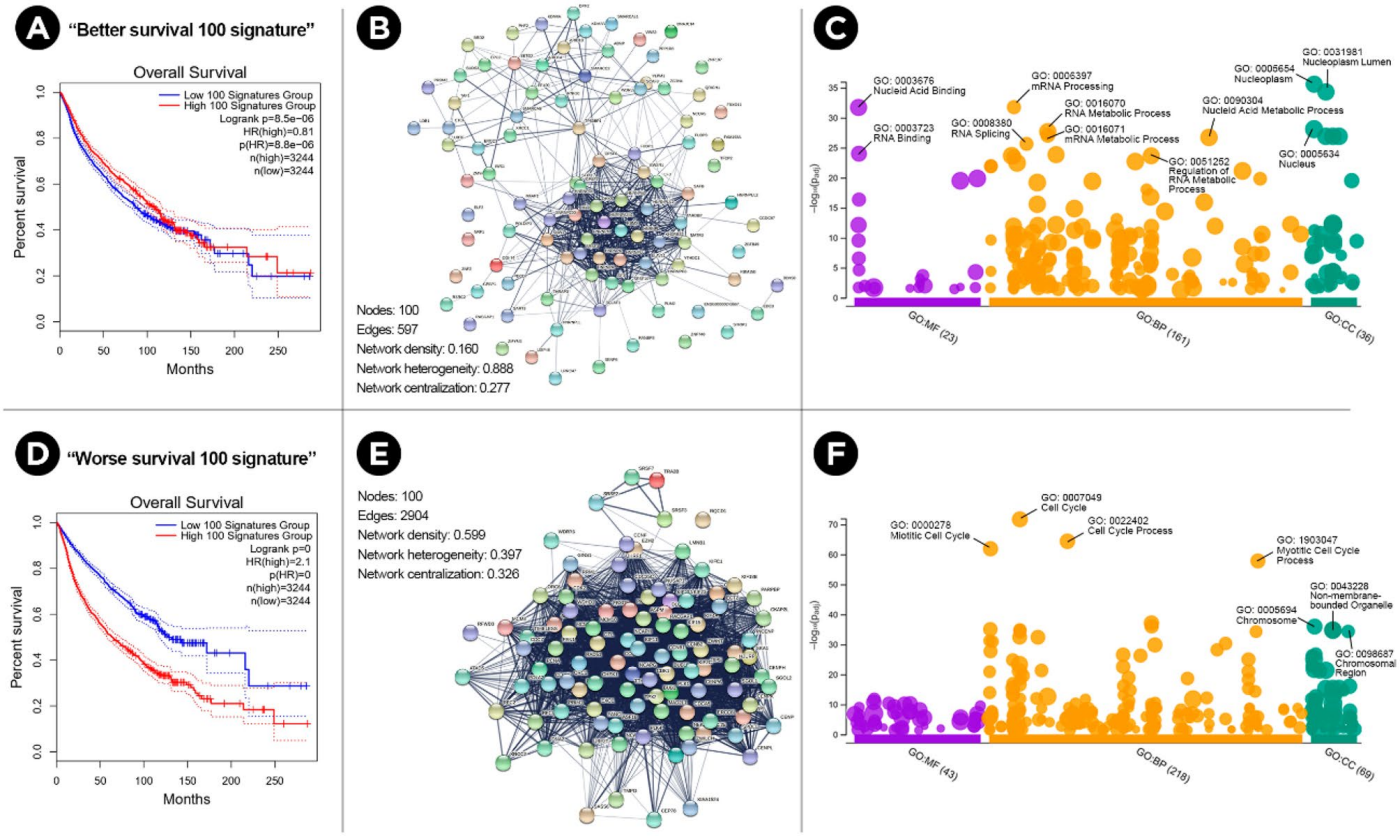
Characterization of the “Better/Worse 100 signature”. (**A**,**D**) Multi-gene signature survival analysis based on the expression of the 100 genes correlated with the “better” and “worse” survival signature (in all 18 cancer types). (**B**,**E**) Protein–protein interaction networks of each multi-gene signature. (**C**,**F**) Gene Ontology enrichment analysis and visualization for each multi-gene signature. GO terms with the lowest adjusted p-value are indicated.

To further understand these molecular signatures, the GEPIA2 web server^21^ (which is also based on TCGA dataset) was used to identify genes with similar expression patterns across the 18 cancer types. For each signature (better/worse), the Pearson’s correlation coefficient of 100 genes was obtained (range 0.65–0.80 for the “better survival” and 0.79–0.84 for the “worse survival”) (Supplementary Table 6). Remarkably, when these genes were used as a multi-gene signature to perform survival analysis based on their expression levels in the 18 cancer types, a higher expression of the “better 100 signature” was found to be also associated with better survival (p = 8.5e−06; HR 0.81; pHR = 8.8e−06; n = 3244; Fig. 7A). On the contrary, a higher expression of the “worse 100 signature” was associated with worse survival (p = 0; HR 2.1; pHR = 0; n = 3244; Fig. 7D).

To further characterize the genes involved in each signature, the STRING web server^22^ was used to plot their protein–protein interaction networks. The stringApp^23^, was used to import the STRING networks into Cytoscape^24^ for network analysis. Overall, the worse survival signature network showed a much higher network density and centralization, as well as lower network heterogeneity (Fig. 7B,E). Additionally, the g:Profiler web server^25^ was used for functional interpretation of these signatures according to their gene ontology (Fig. 7C,F). Interestingly, most of the genes from the “better survival 100 signature" are annotated in functions related to transcription regulation and mRNA metabolism. On the other hand, genes from the "worse survival 100 signature" are annotated in functions related to DNA replication and cell cycle (Supplementary Table 7).

**Figure 8:**
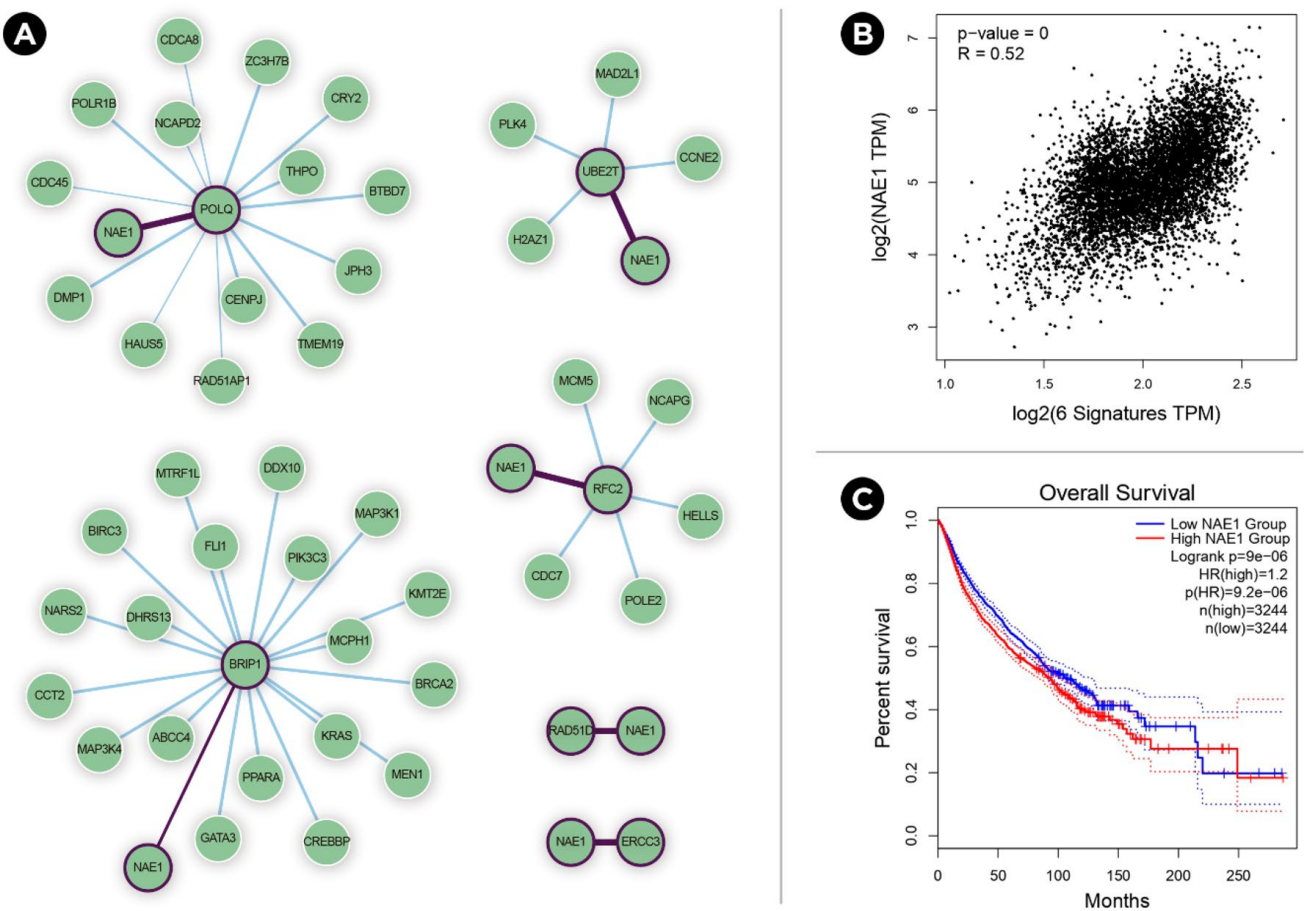
Synthetic lethality gene pairs analysis. (**A**) Synthetic Lethal partners (SLP) were obtained for the 19 genes from the “worse survival” signature. Although NAE1 was the SLP with the highest statistic score in ten on these genes, it was the only significant SLP of six genes. (**B**) The expression levels of these six genes are positively correlated with that of NAE1 in the 18 cancer types. (**C**) Higher expression of NAE1 is statistically significant associated with worse prognosis in the 18 cancer types.

Finally, considering that DDR genes have been good candidates for the synthetic lethality approach^26^ and considering the huge impact of the worse survival signature in terms of overall survival, the SynLethDB^27^ was used to search synthetic lethal partners for the 19 genes from this signature. SynLethDB harbours a large set of synthetic lethality gene pairs collected from a variety of sources, including biochemical assays, computational predictions, other related databases, and text mining. Sixteen genes presented at least one potential synthetic lethal partner (SLP) (statistic score ≥ 0.50). In 10 of these genes (ERCC3, BRIP1, POLQ, UBE2T, RFC2, RAD51D, MUS81, PCNA, RPA3, RFC4), NAE1 (NEDD8 Activating Enzyme E1 Subunit 1) was the SLP with the highest score, and in 6 of them, it was the only gene with a statistic score ≥ 0.50 (Fig. 8A). To further explore this finding, the correlation between the expression levels of these 6 genes and NAE1 was analyzed in GEPIA2. A statistically significant positive correlation was found in the 18 types of cancer (*R* = 0.52, p = 0, *Spearman’s correlation test*, Fig. 8B). In fact, higher expression of NAE1 was also found to be associated with a worse prognosis in all 18 cancer types (p = 9e−06.; HR 1.2; pHR = 9.2e−06; n = 3244; Fig. 8C).

## Discussion

The analysis of cancer dependencies and vulnerabilities aims to identify and prioritize new potential therapeutic targets. In this work, a Pan-cancer analysis of non-oncogene addiction to DDR genes using data derived from the Cancer Dependency Map and TCGA was performed. Among the 59 commonly essential DDR genes, large differences were found between them in terms of dependency scores across the 423 cancer cell lines. However, it must be considered that dependency scores are measured for cells grown in culture and are unlikely to fully reflect the conditions of the in vivo tumour microenvironment. Thus, results must be interpreted with caution and should be further addressed through different approaches.

Since some of the common essential DDR genes in cancer cells may also be essential for normal cells, the implications of essentiality were analyzed at the transcriptomic level from paired normal-tumour samples and large differences were also found. Genes with strongly negative dependency scores (cluster 1), were upregulated in most cancer types suggesting that they might be essential for carcinogenesis. Some of them can even be considered as potential cancer drivers as previously suggested^6^. Despite this, it was surprising that higher expression of several genes from clusters 1 and 2 (e.g., PRPF19 and ATRIP) were strongly associated with better overall survival in some cancer groups. This was further confirmed when the 14 genes exclusively associated with better survival were considered a multi-gene signature and the higher expression of this signature was also associated with better survival across the 18 types of cancer. This is especially interesting considering that the lower expression and/or alterations in DDR genes have been associated with improved survival in some cancer types^28–30^. Nevertheless, a couple of studies have also reported that active DNA repair and higher expression of several DDR genes (including genes from clusters 1 and 2 from this study) were associated with better survival in gastric and ovarian cancer^31,32^. Taken together, this better survival signature might represent promising biomarkers for prognosis and further clinical studies should be performed to fully validate this.

Another surprising finding was that genes from cluster 3 (weakest dependency and efficacy scores) like UBE2T, POLQ, BRIP1, H2AFX, and RTEL1, were also highly upregulated. This could indicate that cancer cells do not depend on these genes for their growth but survival, especially considering the persistent DNA damage and replication stress in cancer cells^2^. We can refer to this as the timing-dependent addiction, which means that although cancer cells have weak dependency on these genes (as growth promoters), these genes may act as late promoters of cell survival (given the evident overexpression). For instance, among genes from cluster 3, POLQ (Pol θ) might be one of the best examples of non-oncogene addiction to DNA repair (low efficacy and dependency scores but highly upregulated). In fact, it has been demonstrated that cancer cells with mutations in POLQ synthetic lethal (DDR) genes tend to become addicted to Theta Mediated End Joining (TMEJ) for survival, suggesting that POLQ becomes essential upon increased levels of endogenous and unrepaired DNA damage^33^.

Remarkably, the present work also demonstrated that higher expression of UBE2T, RFC4, POLQ, BRIP1, and H2AFX is especially associated with worse overall survival (HR > 5) in several types of cancer (Group A and D). Additionally, when considered as part of the worse survival signature, these genes were associated with a worse prognosis in the 18 cancer types. This might be also linked to the correlation found between the expression levels of this signature and the "worse 100 signature", which is enriched with genes involved in DNA replication and cell cycle, probably suggesting a more aggressive phenotype. Previous findings have reported that genes involved in cell cycle phases and checkpoint genes had higher essentiality percentages both in embryonic stem cells and in cancer cell lines^34^. Additionally, it has been shown that the amplification and overexpression of several DDR genes across the pan-cancer scale are associated with reduced mutation burden, cell line drug resistance, and poor prognosis^35^. In contrast, a higher somatic tumour mutational burden has been associated with better overall survival in several types of cancer^36^. Taken together, the results from the present study support the notion that non-oncogene addiction to several DDR genes could reduce the tumour mutational burden and confer treatment resistance, suggesting an explanation for the worse overall survival associated with this signature.

The results discussed above also support previous findings reporting the key role of the DDR genes from cluster 3 in cancer. For instance, studying more than 10,000 TCGA pan-cancer tumours, Wu and colleagues found that tumours with amplifications in DDR genes like UBE2T (Ubiquitin Conjugating Enzyme E2 T) exhibited significantly reduced mutation burden, temozolomide resistance, and worse patient survival. In fact, UBE2T and BRIP1 are also in the top 10 amplified DDR genes in their study^35^. RFC4 (Replication factor C subunit 4) has been also identified as an upregulated DDR gene across the pan-cancer scale^10^. RFC4 protects colorectal cancer cells from X-ray-induced DNA damage and apoptosis through nonhomologous end joining (NHEJ)-mediated DNA repair^37^ and has also been associated with poorer prognosis in this malignancy^38^ and lung cancer^39^.

DNA polymerase theta (Pol θ) (which is encoded by the *POLQ* gene) is important in the repair of genomic double-strand breaks (DSBs) from many sources^40^. Upregulation of POLQ has been associated with poor clinical outcomes in breast and lung cancer^41–44^. Following the same line, the present study highlights the importance of POLQ as a prognostic factor associated with worse survival in different types of cancer (especially Group A and D). Recent reports have demonstrated that POLQ inhibitors selectively kill HR-deficient tumour cells in vitro and in vivo^45,46^. Additionally, it has been demonstrated that POLQ knockdown sensitizes several tumour cell lines to Ionizing Radiation and causes minimal effects on normal tissue radiosensitivity^47^.

RFC2, CHEK1, PCNA, and POLE2 were also associated with worse overall survival in several types of cancer (Group A and D). As they belong to cluster 1 (strongly negative dependency and efficacy scores and high expression levels), these genes might be more necessary for tumour growth and promotion. Upregulation of PCNA has been reported in several types of cancer and its inhibition is gaining more interest as a novel strategy in many cancers^48–50^. Similarly, the knockdown of POLE2 (DNA polymerase epsilon subunit 2) has been linked to reduced or suppressed tumorigenesis in different types of cancer^51–53^. CHEK1 (also known as Chk1 or checkpoint kinase 1) is essential to maintain cell viability in cancer cells^54^ and it has been shown that the combination of ATR and CHEK1 inhibitors results in cancer-specific synthetic lethality^55^. Notably, CHEK1 inhibition is synthetically lethal with loss of B-Family DNA Polymerases like POLE2 in lung cancer and colorectal cancer cells^56^. Taken together, inhibiting these genes from cluster 1 represents a promising therapeutic avenue in several types of cancer, especially focusing on the combined inhibition of CHEK1 and POLE2.

Finally, it was remarkable that NAE1 was identified as a synthetic lethal partner of ten genes from the worse survival signature. NAE1 is responsible for Neddylation, a post-translational modification that adds an ubiquitin-like protein (NEDD8) to multiple substrate proteins and it has been shown that its inhibition exerts anticancer effects mainly by triggering cell apoptosis, autophagy, and senescence^57,58^. In fact, Neddylation is beginning to be considered as a key factor of the DNA repair process^59^. As NAE1 inhibition has been proposed as a new approach to treating cancer^60^, these results support the potential of the combined inhibition of NAE1 and several DDR genes. More research will be needed to validate the implication of the molecular signatures described in this study and to what extent they apply to different subtypes of cancer and the complexity of their genetic backgrounds.

## Methodology

All data processing steps, and statistical analyses were performed in the RStudio 1.4.1717 statistical environment (https://www.rstudio.com/).

### Efficacy, selectivity, and dependency of DNA repair genes

The list of DNA repair genes and their respective pathway annotation was obtained from one of the latest and more complete reports so far^6^. The efficacy and selectivity scores for the 241 genes (θ = 0.6; CRISPR:shRNA = 60%:40%; where θ is the mixing ratio of the two scores) were obtained from shinyDepMap^14^ (https://labsyspharm.shinyapps.io/depmap). These values are derived from a combined CRISPR-shRNA gene dependency score based on the Cancer Dependency Map dataset^11^ (https://depmap.org/portal/). Additionally, for those genes with efficacy scores lower than -0.5 and selectivity scores lower than 0 (termed essential genes), the combined (θ = 0.6; CRISPR:shRNA = 60%:40%, where θ is the mixing ratio of the two scores) dependency scores across 423 cell lines were obtained. Likewise, the lineage-dependent essentiality of 17 cell lineages was also analyzed. For a lineage to be classified as dependent, all its cell lines must have dependency values that exceed the essentiality threshold obtained by analyzing all available genes.

### Transcriptomic alterations of commonly essential DRGs

For transcriptomic analysis, the TACCO (Transcriptome Alterations in CanCer Omnibus) webserver^18^ (http://tacco.life.nctu.edu.tw/) was used to identify the patterns of expression of essential DNA repair genes (efficacy < − 0.5; selectivity < 0) in 18 cancer types. TACCO includes the mRNAs expression levels (Transcripts Per Million) for 26 cancer types from the Broad GDAC Firehose (https://gdac.broadinstitute.org/) (version stddata__2016_01_28). For this task, only the log2 fold change (log2_FC) of those genes with statistically significant (Benjamini-Hochberg) adjusted p values were included. For the heatmap elaboration, the ComplexHeatmap^61^ R package was used.

To find clustering patterns among DDR genes, all variables were analyzed together. The efficacy and selectivity scores were included for each gene as continuous variables. Based on the median dependency score of each gene (for the whole set of 423 cell lines), dependency scores were transformed from continuous to categorical: weakly negative (from 0 to − 0.5), moderate (from − 0.5 to − 1.0), and strongly negative (from − 1.0 to − 1.5). The log2 FC was also transformed to categorical: downregulated (from 0 to − 1.25), upregulated (from 0 to 1.25), and highly upregulated (from 1.25 to 2.5 +). A distance matrix was created based on Gower's similarity coefficient, as it is suggested for mixed-type variables. The clustering algorithm used was Partitioning Around Medoids (PAM) and the silhouette plot was used to determine the number of clusters. The t-Distributed Stochastic Neighbor Embedding (tSNE) algorithm was used for dimensionality reduction (perplexity = 10, max_iter = 1000). Hierarchical clustering was used as an additional algorithm for dimensionality reduction based on the same Gower's similarity coefficients calculated.

### Survival analysis, protein networks, and gene ontology

The GEPIA (Gene Expression Profiling Interactive Analysis) webserver^19^ (http://gepia.cancer-pku.cn/) was used to compare the different gene clusters in terms of patient overall survival across the cancer groups included in this study. GEPIA is based on The Cancer Genome Atlas (TCGA)^20^ and The Genotype-Tissue Expression (GTEx) project^62^. The thresholds for high/low gene expression levels between cohorts were adjusted to 50%. Log-rank P values, hazard ratios (HR), and 95% confidence intervals (CI) were obtained for each gene. Notably, as GEPIA calculations are run in R, some p values might be reported as cero since R does not report values lower than 2.220446e-16 (.Machine$double.eps). Therefore, p values of cero must be understood as “below machine precision”.

The GEPIA2 web server^21^ (http://gepia2.cancer-pku.cn/#index) was used to identify genes that have a similar expression pattern for each signature across all cancer types. The Search Tool for the Retrieval of Interacting Genes/Proteins database (STRING v11.0) web server^22^ (https://string-db.org/) was used to construct the protein–protein network associated with the better/worse 100 signature). The stringApp^23^, a Cytoscape app that makes it easy to import STRING networks into Cytoscape^24^ was used for network visualization and analysis. Additionally, the g:Profiler web server^25^ (http://biit.cs.ut.ee/gprofiler/) was used to find statistically significant Gene Ontology terms, pathways, and other gene functions giving a list of DDR genes.

### Synthetic lethal pair analysis

The SynLethDB web server^27^ (http://synlethdb.sist.shanghaitech.edu.cn/v2/#/) was used to search synthetic lethal partners given a specific gene. SynLethDB harbours a large set of synthetic lethality gene pairs collected from a variety of sources, including biochemical assays, other related databases, computational predictions, and text mining.

## Supplementary Information

Supplementary Table 1.

Supplementary Table 2.

Supplementary Table 3.

Supplementary Table 4.

Supplementary Table 5.

Supplementary Table 6.

Supplementary Table 7.

## References

[1] Weinstein IB, Joe A (2008). Oncogene addiction. Cancer Res..

[2] Luo J, Solimini NL, Elledge SJ (2009). Principles of cancer therapy: Oncogene and non-oncogene addiction. Cell.

[3] Jeggo PA, Pearl LH, Carr AM (2016). DNA repair, genome stability and cancer: A historical perspective. Nat. Rev. Cancer.

[4] Hanahan D, Weinberg RA (2011). Hallmarks of cancer: The next generation. Cell.

[5] Negrini S, Gorgoulis VG, Halazonetis TD (2010). Genomic instability—An evolving hallmark of cancer. Nat. Rev. Mol. Cell Biol..

[6] Knijnenburg TA (2018). Genomic and molecular landscape of DNA damage repair deficiency across The Cancer Genome Atlas. Cell Rep..

[7] Dietlein F, Thelen L, Reinhardt HC (2014). Cancer-specific defects in DNA repair pathways as targets for personalized therapeutic approaches. Trends Genet..

[8] Solimini NL, Luo J, Elledge SJ (2007). Non-oncogene addiction and the stress phenotype of cancer cells. Cell.

[9] Hjaltelin JX (2019). Identification of hyper-rewired genomic stress non-oncogene addiction genes across 15 cancer types. NPJ Syst. Biol. Appl..

[10] Chae YK (2016). Genomic landscape of DNA repair genes in cancer. Oncotarget.

[11] Tsherniak A (2017). Defining a Cancer Dependency Map. Cell.

[12] Meyers RM (2017). Computational correction of copy number effect improves specificity of CRISPR–Cas9 essentiality screens in cancer cells. Nat. Genet..

[13] Behan FM (2019). Prioritization of cancer therapeutic targets using CRISPR–Cas9 screens. Nature.

[14] Shimada K, Bachman JA, Muhlich JL, Mitchison TJ (2021). shinyDepMap, a tool to identify targetable cancer genes and their functional connections from Cancer Dependency Map data. Elife.

[15] Gonçalves E (2020). Drug mechanism-of-action discovery through the integration of pharmacological and CRISPR screens. Mol. Syst. Biol..

[16] Chan EM (2019). WRN helicase is a synthetic lethal target in microsatellite unstable cancers. Nature.

[17] Wood RD, Mitchell M, Lindahl T (2005). Human DNA repair genes, 2005. Mutat. Res. Fundam. Mol. Mech. Mutagen..

[18] Chou P-H (2019). TACCO, a database connecting transcriptome alterations, pathway alterations and clinical outcomes in cancers. Sci. Rep..

[19] Tang Z (2017). GEPIA: A web server for cancer and normal gene expression profiling and interactive analyses. Nucleic Acids Res..

[20] The Cancer Genome Atlas Research Network (2013). The Cancer Genome Atlas Pan-Cancer analysis project. Nat. Genet..

[21] Tang Z, Kang B, Li C, Chen T, Zhang Z (2019). GEPIA2: An enhanced web server for large-scale expression profiling and interactive analysis. Nucleic Acids Res..

[22] Szklarczyk D (2019). STRING v11: Protein–protein association networks with increased coverage, supporting functional discovery in genome-wide experimental datasets. Nucleic Acids Res..

[23] Doncheva NT, Morris JH, Gorodkin J, Jensen LJ (2019). Cytoscape StringApp: Network analysis and visualization of proteomics data. J. Proteome Res..

[24] Shannon P (2003). Cytoscape: A software environment for integrated models of biomolecular interaction networks. Genome Res..

[25] Reimand J (2016). g:Profiler—A web server for functional interpretation of gene lists (2016 update). Nucleic Acids Res..

[26] Nickoloff JA, Jones D, Lee S-H, Williamson EA, Hromas R (2017). Drugging the cancers addicted to DNA repair. JNCI J. Natl. Cancer Inst..

[27] Guo J, Liu H, Zheng J (2016). SynLethDB: Synthetic lethality database toward discovery of selective and sensitive anticancer drug targets. Nucleic Acids Res..

[28] Mo X (2017). Low expression of 12 DNA repair genes was associated with better disease-free survival in non-small cell lung cancer patients having adjuvant chemotherapy. Int. J. Radiat. Oncol. Biol. Phys..

[29] van der Doelen MJ (2020). Impact of DNA damage repair defects on response to radium-223 and overall survival in metastatic castration-resistant prostate cancer. Eur. J. Cancer.

[30] Teo MY (2017). DNA damage response and repair gene alterations are associated with improved survival in patients with platinum-treated advanced urothelial carcinoma. Clin. Cancer Res..

[31] Sun H (2019). Identification of a prognostic signature associated with DNA repair genes in ovarian cancer. Front. Genet..

[32] Jinjia C (2019). The use of DNA repair genes as prognostic indicators of gastric cancer. J. Cancer.

[33] Feng W (2019). Genetic determinants of cellular addiction to DNA polymerase theta. Nat. Commun..

[34] Viner-Breuer R, Yilmaz A, Benvenisty N, Goldberg M (2019). The essentiality landscape of cell cycle related genes in human pluripotent and cancer cells. Cell Div..

[35] Wu Z (2020). Copy number amplification of DNA damage repair pathways potentiates therapeutic resistance in cancer. Theranostics.

[36] Samstein RM (2019). Tumor mutational load predicts survival after immunotherapy across multiple cancer types. Nat. Genet..

[37] Wang X-C (2019). Genome-wide RNAi screening identifies RFC4 as a factor that mediates radioresistance in colorectal cancer by facilitating nonhomologous end joining repair. Clin. Cancer Res..

[38] Xiang J (2014). Levels of human replication factor C4, a clamp loader, correlate with tumor progression and predict the prognosis for colorectal cancer. J. Transl. Med..

[39] Chen W, Zhu S, Zhang Y, Xiao J, Tian D (2019). Identification of key candidate tumor biomarkers in non-small-cell lung cancer by in silico analysis. Oncol. Lett..

[40] Wood RD, Doublié S (2016). DNA polymerase θ (POLQ), double-strand break repair, and cancer. DNA Repair.

[41] Lemee F (2010). DNA polymerase up-regulation is associated with poor survival in breast cancer, perturbs DNA replication, and promotes genetic instability. Proc. Natl. Acad. Sci..

[42] Higgins GS (2010). Overexpression of *POLQ* confers a poor prognosis in early breast cancer patients. Oncotarget.

[43] Shinmura K (2019). POLQ overexpression is associated with an increased somatic mutation load and PLK4 overexpression in lung adenocarcinoma. Cancers.

[44] Allera-Moreau C (2012). DNA replication stress response involving PLK1, CDC6, POLQ, RAD51 and CLASPIN upregulation prognoses the outcome of early/mid-stage non-small cell lung cancer patients. Oncogenesis.

[45] Zhou J (2021). A first-in-class polymerase theta inhibitor selectively targets homologous-recombination-deficient tumors. Nat. Cancer.

[46] Zatreanu D (2021). Polθ inhibitors elicit BRCA-gene synthetic lethality and target PARP inhibitor resistance. Nat. Commun..

[47] Higgins GS (2010). A small interfering RNA screen of genes involved in DNA repair identifies tumor-specific radiosensitization by POLQ knockdown. Cancer Res..

[48] Dillehay KL, Lu S, Dong Z (2014). Antitumor effects of a novel small molecule targeting PCNA chromatin association in prostate cancer. Mol. Cancer Ther..

[49] Smith SJ (2020). Molecular targeting of cancer-associated PCNA interactions in pancreatic ductal adenocarcinoma using a cell-penetrating peptide. Mol. Ther. Oncolytics.

[50] Søgaard CK (2019). Targeting the non-canonical roles of PCNA modifies and increases the response to targeted anti-cancer therapy. Oncotarget.

[51] Li J (2018). Knockdown of POLE2 expression suppresses lung adenocarcinoma cell malignant phenotypes in vitro. Oncol. Rep..

[52] Zhang C (2021). Targeting POLE2 creates a novel vulnerability in renal cell carcinoma via modulating stanniocalcin 1. Front. Cell Dev. Biol..

[53] Zhu Y, Chen G, Song Y, Chen Z, Chen X (2020). POLE2 knockdown reduce tumorigenesis in esophageal squamous cells. Cancer Cell Int..

[54] Cho SH, Toouli CD, Fujii GH, Crain C, Parry D (2005). Chk1 is essential for tumor cell viability following activation of the replication checkpoint. Cell Cycle.

[55] Sanjiv K (2016). Cancer-specific synthetic lethality between ATR and CHK1 kinase activities. Cell Rep..

[56] Rogers RF (2020). CHK1 inhibition is synthetically lethal with loss of B-family DNA polymerase function in human lung and colorectal cancer cells. Cancer Res..

[57] Zhou L, Jiang Y, Luo Q, Li L, Jia L (2019). Neddylation: A novel modulator of the tumor microenvironment. Mol. Cancer.

[58] Chen P (2016). Neddylation inhibition activates the extrinsic apoptosis pathway through ATF4–CHOP–DR5 axis in human esophageal cancer cells. Clin. Cancer Res..

[59] Brown JS, Jackson SP (2015). Ubiquitylation, neddylation and the DNA damage response. Open Biol..

[60] Soucy TA (2009). An inhibitor of NEDD8-activating enzyme as a new approach to treat cancer. Nature.

[61] Gu Z, Eils R, Schlesner M (2016). Complex heatmaps reveal patterns and correlations in multidimensional genomic data. Bioinformatics.

[62] Lonsdale J (2013). The genotype-tissue expression (GTEx) project. Nat. Genet..

